# Just the Things (Poem)

**Published:** 1919-02

**Authors:** Jane Arden


### TEXAS MEDICAL JOURNAL

DR. F. E. DANIEL,
Founder.

Established July,
1885

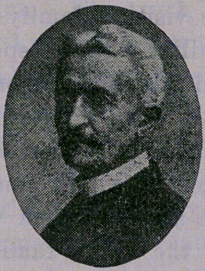

MRS. F. E. DANIEL,
Publisher and Managing
Editor.

VOL. XXXIV No. 8
AUSTIN,
FEBRUARY, 1919.

PUBLISHED MONTHLY.
Subscription:
$1.50

The publisher is not responsible for views of contributors.

ASSISTANT EDITORS:

DR. JOHN S. TURNER, Dallas, Texas.
DR. E. B. PARSONS, Palestine, Texas.
DR. A. P. HOWARD, Houston, Texas.
DR. JOE BECTON, Greenville, Texas.
DR. M. F. BLEDSOE, Port Arthur.
DR. J. H. FLORENCE, Tulsa, Oklahoma.
DR. J. H. REUSS, Cuero, Texas.

### EDITORIAL.

### Just the Things.

Just a golden sunrise, just a word of cheer,
Just a summer shower, just a rainbow clear,
Just a crimson sunset, just a purple hill,
Just a shaft of moonlight when the world is still,
Just a little fragrant breeze, just an azure sky;
Just the murmur of the sea, just a baby's cry;
Just a nodding flower, just a bird's sweet lay,
Just someone to greet us at the close of day,
Just a haunting melody—a half-forgotten song,
Just the glad warm sunshine through the whole day long;
Just the doing of our bit ever willingly,
Just the chance of helping—those across the sea,
Just a friendly handclasp, just a word of love,
Just the simple knowledge God is there above;
Just an act of kindness, just a sunny smile—
These are just the things that make our life worth while.

—Jane Arden in the *People's Home Journal*.

